# Pseudohypoxemia From Leukocyte Larceny in a Patient With Chronic Myelogenous Leukemia

**DOI:** 10.7759/cureus.21405

**Published:** 2022-01-19

**Authors:** Saeid Mirzai, Mark Andreae, Chethan Puttarajappa

**Affiliations:** 1 Department of Medicine, Alabama College of Osteopathic Medicine, Dothan, USA; 2 Department of Critical Care Medicine, University of Pittsburgh, Pittsburgh, USA; 3 Department of Medicine, University of Pittsburgh, Pittsburgh, USA

**Keywords:** pulse oximetry, arterial blood gas, false hypoxemia, leukemia, leukocyte larceny

## Abstract

Arterial blood gas (ABG) analysis is a generally reliable and frequently employed test for evaluating blood oxygen content. False readings of low oxygen content are rare but can be expected in specific clinical scenarios such as leukemia patients with marked leukocytosis who can develop “leukocyte larceny,” a phenomenon of excess oxygen consumption by leukocytes. Awareness of this phenomenon may lead to early recognition and avoidance of unnecessary diagnostic and therapeutic interventions. This case report presents a patient with marked leukocytosis from chronic myelogenous leukemia whose extubation was briefly delayed due to pseudohypoxemia on ABG measurements.

## Introduction

Arterial blood gas (ABG) analysis is commonly used to diagnose and manage disorders of oxygenation, ventilation, and acid-base balance. However, false abnormal results do occur despite their accuracy and reliability [1].

One such phenomenon occurs with delays in the analysis of arterial blood following collection, which can lead to a fall in arterial oxygen tension (PaO_2_). These changes are primarily due to the continued metabolic activity of blood cells in the sample [2]. With normal cell counts, this is insignificant. However, in patients with leukemia or thrombocytosis, rapid oxygen consumption by the many cells present in the sample can give false readings [3].

This little-known concept could help avoid unnecessary testing and alterations in management, leading to better outcomes in this patient population. Here we present a patient suffering from chronic myelogenous leukemia (CML) whose extubation was briefly delayed due to falsely low (partial pressure of oxygen) PaO_2_ readings on ABG analysis.

## Case presentation

A 40-year-old male with a history of BCR-ABL1-positive CML and non-compliance with imatinib was admitted to the hospital with hemoperitoneum from non-traumatic splenic rupture. On admission, he was tachycardic (108 beats/minute) and tachypneic (32 breaths/minute) with a blood pressure of 128/88 mmHg and oxygen saturation of 97% via pulse oximetry on ambient air. He had a distended and diffusely tender abdomen but an otherwise unremarkable cardiopulmonary exam. Labs showed a hemoglobin of 6.6 g/dL, white blood cell (WBC) count of 485,000/mm^3^ with expanded myelogenous precursors, pseudo-hyperkalemia with a peak of 9.4 mMol/L, and normal platelet counts.

He underwent an emergent exploratory laparotomy with splenectomy. Intra-operatively and during the post-operative intensive care unit stay, he was noted to have persistent arterial hypoxemia with PaO_2_ ranging from 51 to 61 mmHg and oxygen saturations between 82% to 89% on multiple ABG measurements. His bedside continuous pulse oximetry, however, showed oxygen saturations between 95% to 100% during the same time (Table 1). Carboxyhemoglobin was only mildly elevated at 1.7% to 2.2%. Chest radiographs were normal.

**Table 1:** Oxygenation readings during the hospital course PaO_2_, SaO_2_, PaCO_2_, and pH represent arterial blood gas readings whereas SpO_2_ was measured via pulse oximetry. ICU: intensive care unit; PaO_2_: arterial oxygen tension; SaO_2_ and SpO_2_: oxygen saturation; PaCO_2_: arterial carbon dioxide tension; FiO_2_: fraction of inspired oxygen; NC: nasal cannula. ^a^ Data not available.

Hospital course	PaO_2_ (mmHg)	SaO_2_ (%)	PaCO_2_ (mmHg)	pH	SpO_2_ (%)	Inspired oxygen
Intra-operative	59	84	48	7.32	96	40% FiO_2_
ICU (intubated)	51	84	48	7.42	97	40% FiO_2_
ICU (intubated)	61	88	49	7.42	94	40% FiO_2_
ICU (intubated)	60	89	52	7.40	94	40% FiO_2_
ICU (extubated)	53	… ^a^	46	7.46	96	6 L/min NC

The observed discrepancy in oxygen saturation, and lack of cardiopulmonary cause for hypoxemia, briefly delayed extubation plans. However, after a detailed clinical assessment and literature review, the team concluded that the hypoxemia on ABG was possibly due to the marked leukocytosis, and the patient was extubated uneventfully. Imatinib was restarted along with hydroxyurea, and his leukocytosis improved over the ensuing weeks.

## Discussion

ABGs are valuable in managing critically ill patients, and awareness of conditions leading to falsely abnormal values is necessary for the appropriate interpretation of ABG results. Evaluation of pseudohypoxemia should consider common causes such as erroneous venous sampling, ABG analyzer malfunction, and significant delays in specimen transport [1]. Delays in ABG analysis do not significantly affect pH or the partial pressure of carbon dioxide but can lead to a noticeable fall in PaO_2_. These changes are primarily due to the continued metabolic activity of leukocytes, platelets, and reticulocytes in the sample [4]. With normal cell counts, this reduction in PaO_2_ is small, with studies showing a change of -1.05 kPa (approximately -7.9 mmHg) in samples stored at room temperature for 20 minutes [5].

Noticeable decreases in PaO_2_ are found when WBC counts exceed approximately 50,000/mm^3^, a laboratory phenomenon termed “leukocyte larceny” by Fox et al. in 1979 [6-7]. The rate of oxygen consumption is dependent upon leukocyte type and maturity. Monocytes have the highest oxygen consumption rate, followed by granulocytes and lymphocytes [4]. Several studies have shown that leukemic cells have higher rates of oxygen consumption overall [4]. These patients can also have additional false abnormalities such as low glucose readings due to increased glucose utilization and pseudo-hyperkalemia due to damage and cell lysis [7].

Patients with extreme leukocytosis are at risk for both true and false hypoxemia; thus, a rapid and accurate diagnosis is paramount [1]. True hypoxemia can be from pneumonia, pulmonary embolism, and leukostasis in the pulmonary microvasculature causing severe ventilation-perfusion mismatch [1]. These diagnoses require the timely initiation of specific therapies. Pulmonary leukostasis may require plasmapheresis in severe cases [1]. Early recognition of false hypoxemia can avoid medical error and subsequent morbidity from unnecessary interventions.

In situations with marked leukocytosis or thrombocytosis (causing “platelet larceny”), oxygen saturations obtained by ABG should be interpreted with caution [3]; pulse oximetry is the more accurate method of assessing oxygenation in such patients [4]. ABG analysis calculates oxygen saturation from PaO_2_, a measure of dissolved oxygen in plasma. In contrast, pulse oximetry measures oxygen saturation directly from hemoglobin saturation and is thus unaffected by leukocytosis [7]. It is important to ensure the absence of carboxyhemoglobinemia and methemoglobinemia for accurate interpretation (Table 2) [1].

**Table 2:** Conditions altering oxygenation readings PaO_2_ and SaO_2_ represent arterial blood gas (ABG) readings whereas SpO_2_ is measured via pulse oximetry. PaO_2_: arterial oxygen tension; SaO_2_ and SpO_2_: oxygen saturation. ^a^ Co-oximetry can be used to detect carboxyhemoglobin and methemoglobin. ^b^ SaO_2_ would be low if directly measured with newer ABG analyzers rather than derived from PaO_2_. ^c^ Methemoglobin interferes with light absorption at both wavelengths (for oxy- and deoxy-hemoglobin) used by oximeters, thus causing interference with the assessment of oxygen saturation.

Laboratory test	True hypoxemia	Leukocyte larceny	Carboxy-hemoglobinemia ^a^	Met-hemoglobinemia ^a^
PaO_2_	Low	Low	Normal	Normal
SaO_2_	Low	Low	Normal ^b^	Normal ^b^
SpO_2_	Low	Normal	Normal	Low ^c^

There are additional methods for evaluating false hypoxemia on ABGs when larceny is suspected. Continuous ABG sampling could reduce delays in analysis, but the reliability and cost-effectiveness of this method have not been established. Additionally, ABG can be performed on plasma rather than whole blood in patients with leukemia [1]. Attempts at slowing or inhibiting leukocyte metabolism can also be employed. Such techniques include immediately chilling the sample to 2°C until analysis, although results have been inconsistent, or adding sodium fluoride or potassium cyanide to the sample [1,4]. Improved measurement of PaO_2_ can be expected following a reduction in cell counts via chemotherapy or leukapheresis [1].

## Conclusions

In summary, patients with marked leukocytosis can have false hypoxemia on ABG analysis. This phenomenon, termed leukocyte larceny, can be seen when WBC counts exceed approximately 50,000/mm^3^. Therefore, pulse oximetry may be the more accurate method of assessing oxygenation in situations with marked leukocytosis or thrombocytosis. Awareness of this larceny phenomenon may lead to timely recognition of pseudohypoxemia and avoid unnecessary diagnostic and therapeutic measures to correct hypoxemia such as diuretics, antibiotics, changes to ventilator settings, and longer intubation times.
